# Epigenetic modification of TLR4 promotes activation of NF-κB by regulating methyl-CpG-binding domain protein 2 and Sp1 in gastric cancer

**DOI:** 10.18632/oncotarget.6549

**Published:** 2015-12-10

**Authors:** Tae Woo Kim, Seon-Jin Lee, Byung Moo Oh, Heesoo Lee, Tae Gi Uhm, Jeong-Ki Min, Young-Jun Park, Suk Ran Yoon, Bo-Yeon Kim, Jong Wan Kim, Yong-Kyung Choe, Hee Gu Lee

**Affiliations:** ^1^ Genome Structure Research Center, Korea Research Institute of Bioscience and Biotechnology, Yuseong-gu, Daejeon, Republic of Korea; ^2^ Department of Biomolecular Science, University of Science and Technology (UST), Yuseong-gu, Daejeon, Republic of Korea; ^3^ Functional Genomics Research Center, Korea Research Institute of Bioscience and Biotechnology, Yuseong-gu, Daejeon, Republic of Korea; ^4^ Immunotherapy Research Center, Korea Research Institute of Bioscience and Biotechnology, Yuseong-gu, Daejeon, Republic of Korea; ^5^ World Class Institute, Korea Research Institute of Bioscience and Biotechnology, Ochang, Cheongwon, Republic of Korea; ^6^ Department of Laboratory Medicine, College of Medicine, Dankook University, Cheonan, Chungnam, Republic of Korea

**Keywords:** toll-like receptor 4, gastric cancer, Sp1, methyl-CpG-binding domain protein 2, methylation

## Abstract

Toll-like receptor 4 (TLR4) is important in promoting the immune response in various cancers. Recently, TLR4 is highly expressed in a stage-dependent manner in gastric cancer, but the regulatory mechanism of *TLR4* expression has been not elucidated it. Here, we investigated the mechanism underlying regulation of *TLR4* expression through promoter methylation and histone modification between transcriptional regulation and silencing of the *TLR4* gene in gastric cancer cells. Chromatin immunoprecipitation was carried out to screen for factors related to *TLR4* methylation such as MeCP2, HDAC1, and Sp1 on the *TLR4* promoter. Moreover, DNA methyltransferase inhibitor 5-aza-deoxycytidine (5-aza-dC) induced demethylation of the *TLR4* promoter and increased H3K4 trimethylation and Sp1 binding to reactivate silenced TLR4. In contrast, although the silence of *TLR4* activated H3K9 trimethylation and MeCP2 complex, combined treatment with TLR4 agonist and 5-aza-dC upregulated H3K4 trimethylation and activated with transcription factors as Sp1 and NF-κB. This study demonstrates that recruitment of the MeCP2/HDAC1 repressor complex increases the low levels of TLR4 expression through epigenetic modification of DNA and histones on the *TLR4* promoter, but Sp1 activates TLR4 high expression by hypomethylation and NF-κB signaling in gastric cancer cells.

## INTRODUCTION

Toll-like receptor 4 (TLR4) is an important member of type I transmembrane protein family. TLR4 is expressed not only on immune cells but also on cancer cells such as cervical cancer [1–3] and lung cancer [4]. Although the TLR4 profile varies in different tumor cells, current evidence indicates that the expression of TLR4 and signaling cascade are involved in tumor growth, progression and invasion [5]. For example, TLR4 signaling increased COX-2 and PGE2 signaling and early colorectal carcinogenesis, inhibited apoptosis and promoted angiogenesis [6]. TLR4-mediated cancer growth involved in breast tumor progression and downregulation of TLR4 prevented breast cancer progression and survival [7]. TLR4 expressed on human lung cancer cells is functionally active, and may play important roles in promoting immune escape of human lung cancer cells by inducing immunosuppressive cytokines and apoptosis resistance [8]. TLR4 acts as a functional receptor in the pre-metastatic phase in pulmonary metastasis [9]. These reports implied that TLR4 are expressed on human tumor cells and may play important roles in the progression of cancer.

A recent study suggests that *TLR4* expression is suppressed by epigenetic events *via* methylation of DNA and histone, which has been recognized as a critical mechanism modulating *TLR4* expression [10]. Epigenetic modification of CpG islands within gene promoters plays a major role in cancer [11,12]. Moreover, methylation of the *TLR4* promoter is associated with *TLR4* silencing in a variety of cells, including intestinal epithelial and stem-cell–derived vascular cells [13,14], but the precise epigenetic mechanism underlying progression of gastric cancer is not fully understood. Silencing by DNA methylation consists of two main mechanisms, *de novo* methylation and methylation mediated by transcription factor binding; the latter inhibits gene expression *via* repressor binding to methylated CpG sites [15]. Following methylation, methyl-CpG-binding domain (MBD) proteins, such as MBD1, MBD2, MBD3, and methyl-CpG binding protein 2 (MeCP2) are commonly recruited to the CpG site, repress transcription by recruiting Sin3A, which interacts with histone deacetylases (HDACs), and form a corepressor complex [16]. Transcription factor Sp1 binds to the *TLR4* promoter [17]. Sp1 binding to the promoters of target genes is often associated with prevention of CpG methylation and acts as an activator [18]. Consistent with this, we hypothesize that methylation of cytosine in CpG islands within Sp1 binding sites may directly suppress to Sp1 binding ability. In addition, we suggest that the differential DNA methylation of the *TLR4* promoter in gastric cancer cell lines expressing *TLR4* at high and low levels is an important mechanism underlying *TLR4* transcription, with TLR4-silenced cells showing increased MeCP2 binding and *TLR4*-upregulated cells showing enhanced Sp1 binding, to the TLR4 promoter.

## RESULTS

### *TLR4* mRNA and protein expression in gastric cancer cell lines and tissues

**Figure 1:**
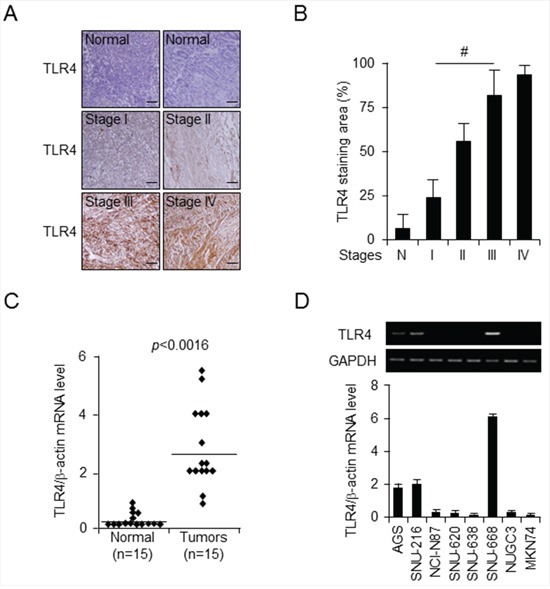
*TLR4* expression pattern in gastric tumors **A.** and **B.** Representative images (A) and quantitation (B) of immunohistochemical analysis of *TLR4* expression in tissue array of pairs (*n* = 55) of normal and gastric cancer tissues at indicated stages (stage I, *n* = 14; stage II, *n* = 14; stage III, *n* = 24; and stage IV, *n* = 3). Original magnification, 200×; scale bars, 50 μm. Statistical significance was calculated using a *t*-test; #, *p* < 0.01. **C.** *TLR4* mRNA expression was monitored by quantitative PCR in 15 paired gastric normal and tumor tissues from gastric cancer patients. **D.** qPCR and reverse transcription-PCR analysis of *TLR4* mRNA expression level was carried out for the indicated gastric cancer cell lines.

To explore potential *TLR4* expression in gastric cancer, immunohistochemical analysis of TLR4 on a tissue array consisting of 55 sets of gastric cancer tissues and adjacent normal tissues was carried out each of tumor stages I and II, 24 cases of stage III, and 3 cases of stage IV. We observed that TLR4 was expressed at a high level in cancer tissue compared to normal tissue, and level was dependent on tumor stage (Figure 1A). Quantification of staining intensity using by Image J (http://openwetware.org/wiki/Sean_Lauber:ImageJ Threshold_Analysis) demonstrated that 20% of the area of tumor stage I samples, 55% in tumor stage II, 80% in tumor stage III, and 90% in tumor stage IV were TLR4-positive, compared to 10% in normal tissue (Figure 1B). Further quantification of TLR4 staining intensity in these tissues revealed that *TLR4* expression was elevated in gastric cancer tissues (70%), but not normal tissues (10%) (data not shown). Furthermore, we confirmed *TLR4* mRNA level in 15 pairs of gastric cancer tissue and adjacent normal tissue using quantitative PCR (qPCR) and found 5-fold enhancement in gastric cancer tissues (*p* < 0.0016) (Figure 1C). We next examined *TLR4* mRNA level in eight gastric cancer cell lines using qPCR and reverse transcription-PCR. Interestingly, *TLR4* mRNA expression was overwhelmingly low levels in all gastric cancer cell lines and high level in SNU-668, AGS, and SNU-216 (Figure 1D). The MethHC database (http://MethHC.mbc.nctu.edu.tw) shows that *TLR4* is expressed through promoter demethylation, but is occasionally downregulated by hypermethylation in gastric cancer tissues. This *in silico* data suggest that *TLR4* expression may be regulated by an epigenetic mechanism, and we hypothesized that *TLR4* expression may be silenced by promoter methylation in gastric cancer cell lines. Taken together, these data indicate that *TLR4* is overexpressed in gastric cancer and suggest the possibility that TLR4 may be a useful biomarker and therapeutic target for gastric cancer, but the precise molecular mechanism underlying regulation of *TLR4* transcription is unclear.

### *TLR4* silencing by promoter methylation in gastric cancer cell lines

**Figure 2:**
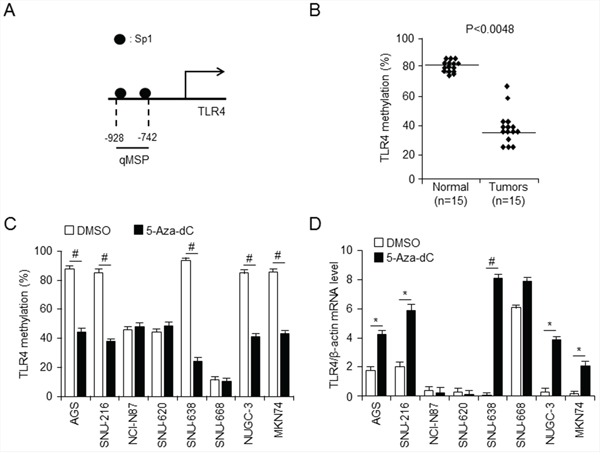
Analysis of relationship between TLR4 expression and promoter methylation in gastric cancer **A.** Schematic diagram of *TLR4* promoter, showing positions of Sp1 binding sites. quantitative methylation-specific PCR (qMSP) were analyzied for methylation pattern of CpG sites on *TLR4* promoter in normal and gastric cancer tissues (*p* < 0.0048) **B.** and the indicated gastric cancer cell lines **C.** treated with 5-aza-dC or DMSO (control) (#*p* < 0.001). The *TLR4* promoter was methylated at a much higher level in low level of *TLR4* mRNA expressing gastric cancer cell line SNU638, but not in SNU668. **D.** qPCR analysis effect of 5-aza-dC on *TLR4* expression in the indicated gastric cancer cells. * *p* < 0.05, # *p* < 0.01. Low level of *TLR4* mRNA expressing SNU638 gastric cancer cells was demethylated by 5-aza-dC.

Following promoter analysis using the program TF Search (http://www.cbrc.jp/research/db/TFSEARCH.html) to identify candidate regulators on the TLR4 gene, binding sites of transcription factor Sp1 were identified on the *TLR4* promoter region (Figure 2A). It is well known that promoter methylation generally interferes with Sp1 binding activity [19]. Demethylation of promoter CpG sites is a key component of epigenetic regulation, and transcription factors, such as Sp1 and Sp3, are required for demethylation and gene expression [20]. We further investigated the methylation status of putative Sp1 binding sites (CCCGCC), including a CpG site on the *TLR4* promoter using quantitative methylation-specific PCR (qMSP) in gastric normal and cancer tissues. The Sp1 binding site was generally hypomethylated in 15 gastric cancer tissues (38%) compared to normal tissues (80%) (Figure 2B) and in gastric cancer cell lines (Figure 2C), and these results were associated with a high level of *TLR4* mRNA expression (Figure 2D). We used qMSP to assess the extent of methylation of the *TLR4* promoter in gastric cancer cell lines expressing low levels of TLR4 and observed that these cells showed high levels of DNA methylation compared with SNU-668 cells, which express a high level of TLR4 (Figure 2C and 2D). However, no change in methylation level of the *TLR4* promoter was observed upon treatment of SNU-668 cells with DNA methyltransferase inhibitor 5-aza-deoxycytidine (5-aza-dC), indicating that the *TLR4* promoter is hypomethylated. Epigenetic events, such as DNA methylation and histone modification, frequently induce silencing of gene transcription [21]. Both 5-aza-dC and trichostatin A (TSA) are often used to reverse silencing by promoter methylation. Therefore, we investigated the effect of 5-aza-dC on promoter methylation (Figure 2C) and of 5-aza-dC and TSA on expression of TLR4 (Figure 2D and Supplementary Figure S1A) in gastric cancer. Consistent with the basal methylation level in gastric cancer cell lines, the CpG island in the Sp1 binding site of the *TLR4* promoter was dramatically demethylated by treatment with 1 μM 5-aza-dC of gastric cancer cell lines expressing low levels of TLR4 in AGS, SNU-216, SNU-668, NUGC-3, and MKN-74 cells (Figure 2C), accompanied by a dramatic increase in *TLR4* mRNA levels in these cells (Figure 2D). However, *TLR4* mRNA level was increased by treatment with 100 nM TSA only in SNU-638 and NUGC-3 cells (Supplementary Figure S1A). Another toll-like receptor, TLR2, also induces NF-κB signaling, similar to TLR4 [22]. Moreover, although TLR4 is associated with LPS-induced signaling, recent reports suggest that TLR2 is located upstream of TLR4 in this pathway [23]. We additionally confirmed basal expression level of TLR2 and effect of 5-aza-dC in gastric cancer cell lines using qPCR, but TLR2 expression level was not affected by 5-aza-dC except SNU-668 cells (Supplementary Figure S1B). These results imply that *TLR4* expression is strongly associated with promoter methylation status, which may be one of the most important factors to regulate *TLR4* expression in gastric cancer cell lines.

### *TLR4* expression is regulated through DNA and histone methylation at the Sp1 binding site

**Figure 3:**
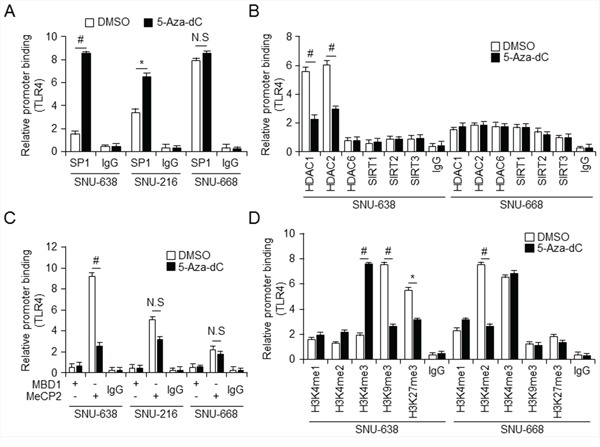
Relative relationship between Sp1 and MeCP2 binding to *TLR4* promoter **A–D.** Chromatin immunoprecipitation (ChIP) was performed with lysates of control (DMSO-) and 5-aza-dC–treated SNU638, SNU216, and SNU668 gastric cancer cells and (control) IgG and antibodies against Sp1 (A); HDAC1, HDAC2, HDAC6, SIRT1, SIRT2, SIRT3 (B); MBD1, MeCP2 (C); and H3K4me1, H3K4me2, H3K4me3, H3K9me3, and H3K27me3 (D). * *p* < 0.05, # *p* < 0.01.

Based on the results shown in Figure 2, we proposed that silencing of *TLR4* is correlated with hypermethylation. Transcription factors, such as Sp1 and CTCF, are recruited to and suppressed CpG sites in promoters from DNA methylation, activating gene expression, whereas HDAC activity and MBD proteins are transcriptional repressors [24,25]. To determine whether methylation or lack of methylation at the CpG site on the *TLR4* promoter influences Sp1 binding, we first carried out quantitative chromatin immunoprecipitation (qChIP) to identify the Sp1 binding site on the *TLR4* promoter in SNU-638, SNU-216, and SNU-668 gastric cancer cells which show differential expression of *TLR4* mRNA as shown Figure 2D (Figure 3A). This experiment suggested that Sp1, a transcriptional activator, was able to bind the *TLR4* promoter in SNU-668 cells, which express *TLR4* at a high level, compared with the hypermethylated *TLR4* promoter in the SNU-638 cells. Treatment of SNU-638 cells with 5-aza-dC demethylated the *TLR4* promoter and led to increased binding of Sp1. Subsequently, to investigate whether HDACs, known to be repressors of transcription, were related to binding of Sp1, qChIP was performed with antibodies targeting the HDACs such as HDAC1, 2, and 6 and SIRT 1, 2, and 3 in SNU-216, SNU-638, and SNU-668 cells. qChIP analysis demonstrated that HDAC1 and HDAC2 participate in strong binding of Sp1 in SNU-216 and SNU-638 cells, in contrast to SNU-668 cells, and 5-aza-dC–induced demethylation of the Sp1 site blocks binding of HDAC1 and HDAC2 (Figure 3B and Supplementary Figure S2A). Moreover, the effect of treatment of gastric cancer cells with HDAC inhibitor TSA was similar to that of 5-aza-dC with regard to differential HDAC1 and HDAC2 binding (Supplementary Figure S2B and S2C). The recruitment of HDACs is associated with MBD proteins, which are known to be transcriptional co-repressors [26]. MBD proteins known to be transcription repressors, MBD1 and MeCP2 recruit HDACs to methylated DNA [27]. Binding of MBD1 and MeCP2 to the *TLR4* promoter was also analyzed using qChIP with primers for the Sp1 binding site in gastric cancer cells (Figure 3C). qChIP analysis using antibodies targeting MBD1 and MeCP2 indicated that MeCP2 binds to the Sp1 binding site on the *TLR4* promotor in low level of *TLR4* expressing SNU-638 cells, but not to the hypomethylated promoter in SNU-668 cells, and 5-aza-dC treatment did not lead to MeCP2 binding to the demethylated *TLR4* promoter in gastric cancer cells. MeCP2 binding to the methylated CpG site in the *TLR4* promoter results in recruitment of corepressors, such as HDAC1 and HDAC2, and transcriptional repression. Moreover, MeCP2 cooperates in maintenance of a repressive chromatin state by acting as a link between DNA and histone methylation [28]. When we analyzed ChIP-seq profiles for methylated histones H3K4me3, H3K9me3, and H3K27me3 in gastric primary tissues (in the NCBI Gene Expression Omnibus repository under accession numbers GSM910579, GSM910585, and GSM910561), the level of trimethylated H3K4 in the proximal promoter region of *TLR4* was elevated compared to the levels of trimethylated H3K9 and H3K27. (Epigenome: ChIP-Seq Analysis of H3K9me3 in Human Gastric Tissue; renlab.H3K9me3.STL001GA.01.01). To test whether trimethylation of H3K4, H3K9, and H3K27 is affected by TLR4 mRNA expression, we carried out qChIP of the Sp1 binding site using histone methylation-related antibodies in SNU-638 and SNU-668 cells, which differentially express TLR4. We found that 5-aza-dC and TSA treatments dramatically increased binding of H3K4me3 in SNU-638 cells, which express TLR4 at a low level, and profoundly decreased levels of H3K9me3 and H3K27me3 binding (Figure 3D and Supplementary Figure S3). In contrast, H3K4me3 bound more strongly to the hypomethylated Sp1 binding site in SNU-668 cells than trimethylated H3K9 and H3K27, and this binding was not affected by 5-aza-dC or TSA. Taken together, these data demonstrate a complex epigenetic mechanism associated with differential expression of *TLR4*. These findings also suggest that Sp1 and binding of trimethylated H3K4 to the *TLR4* promoter act as activators of hypomethylated *TLR4* expression, but binding of MeCP2, H3K9me3, and H3K27me3 form a repressor complex and silence *TLR4*.

### Epigenetic regulation of *TLR4* is mediated by MeCP2 and Sp1 *via* HDACs

**Figure 4:**
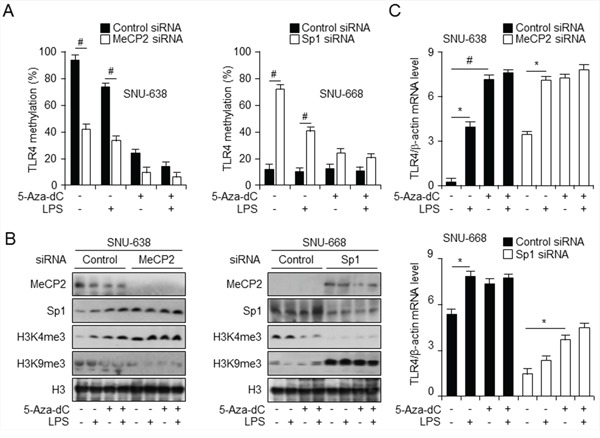
Knockdown of Sp1 and MeCP2 regulate TLR4 expression through modification of DNA and histone methylation **A.** qMSP analysis of CpG sites in *TLR4* promoter in gastric cancer cells transfected with control short interfering RNA (siRNA) and siRNA targeting MeCP2 (left) or Sp1 (right) and treated with lipopolysaccharide (LPS) and 5-aza-dC. # *p* < 0.01. **B.** Western analysis of the indicated proteins in nuclear fraction of cells described in (A) to monitor modifications in methylation of histones. **C.** qPCR analysis of *TLR4* expression level, normalized to that of β-actin, in cells described in (A). *p < 0.05, #*p* < 0.01.

MBD proteins, such as MeCP2, MBD1, and MBD2, together with histone modifications, can repress transcription from methylated promoters [29]. To further explore the mechanism of transcriptional regulation at the *TLR4* promoter, we performed short interfering RNA (siRNA)-mediated knockdown of MeCP2 and Sp1 in SNU638 and SNU668 cells. Cells were then treated with LPS and/or 5-aza-dC alone or together, followed by assessment of cell viability, and qMSP and Western analysis. No difference in cell viability was observed between MeCP2 and Sp1 knockdown and control siRNA-treated cells or between any cells treated with 5-aza-dC and/or LPS (Supplementary Figure S4). In SNU-638 cells, MeCP2 level, decreased by MeCP2 knockdown compared to control siRNA-treated cells, was also downregulated by treatment with LPS and 5-aza-dC alone and together, whereas H3K9me3 level increased with LPS and 5-aza-dC treatment (Figure 4B). Moreover, Sp1 knockdown also resulted in a decrease in H3K4me3, whereas MeCP2 and H3K9me3 levels increased and were not so much different effects with LPS and 5-aza-dC treatment. Interestingly, MeCP2 downregulation contributes to *TLR4* activation and upregulation with LPS and 5-aza-dC treatment in SNU-638 cells, but SNU-668 Sp1 knockdown cells downregulate *TLR4* expression, which was also reduced by LPS and 5-aza-dC treatment, compared to control siRNA-transfected cells (Figure 4C). Together, these findings suggest that MeCP2 regulates H3K9 trimethylation with *TLR4* activation, but Sp1-mediated *TLR4* activation contributes to H3K4 trimethylation. Interestingly, MeCP2 binding following treatment with 5-aza-dC or LPS was associated with activator Sp1 and H3K4me3. MeCP2 regulates H3K9 trimethylation with *TLR4* activation, but Sp1-mediated *TLR4* activation contributes to H3K4 trimethylation.

### Combination 5-aza-dC and LPS treatment enhances binding activity of Sp1 and H3K4me3 in gastric cancer cells *via* activation of TLR4 signaling

**Figure 5:**
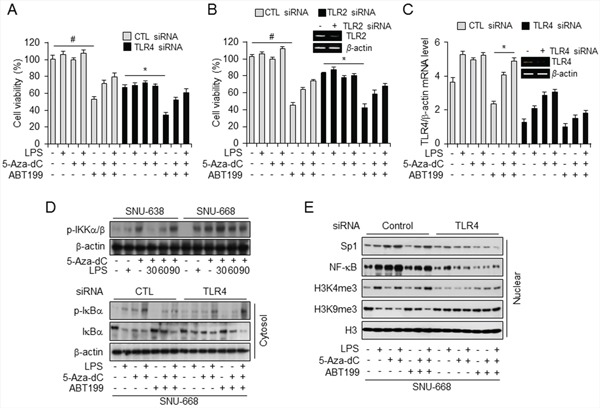
ABT-199 treatment decreased, and combined treatment with LPS and 5-aza-dC restored, TLR4 expression and cell viability **A–C.** Analysis of cell viability (A and B) and *TLR4* mRNA level (C) in SNU668 cells transfected with control siRNA and siRNA targeting *TLR4* and *TLR2,* and treated with LPS, 5-aza-dC, and ABT-199 alone and in the indicated combinations. *p < 0.05, #*p* < 0.01. **D.** Western analysis of p-IKKα/β in SNU638 and SNU668 cells, untreated or pretreated with 5-aza-dC and incubated with LPS for the indicated time (minutes) (top) and of total and phosphorylated IκBα in the cytosolic fraction of SNU668 cells transfected with control siRNA or siRNA targeting *TLR4* and treated with LPS, 5-aza-dC, and ABT-199 alone and in the indicated combinations. β-actin served as internal control. **E.** Western analysis of Sp1, NF-κB, H3K4me3, H3K4me9, and H3 in the nuclear fraction of SNU668 cells transfected with control siRNA or siRNA targeting *TLR4* and treated with LPS, 5-aza-dC, and ABT-199 alone and in the indicated combinations.

To identify whether combination treatment with 5-aza-dC and LPS mediated NF-κB signaling and triggered cell death in *TLR4* (Figure 5A) and *TLR2* (Figure 5B) knockdown cells treated with BCL2 inhibitor ABT199, we first carried out siRNA-mediated knockdown of *TLR4* and *TLR2* in SNU-668 cells, which express TLR4 at a high level, treated the cells with 5-aza-dC and LPS and then with ABT-199, and monitored cell viability. Interestingly, viability was significantly reduced only in cells treated with ABT-199 alone, but viability of ABT-199–treated control and *TLR4* knockdown cells increased following 5-aza-dC and LPS pretreatment. However, silencing effect of TLR2 was similar with the cell viability of TLR4 knockdown in SNU-668. Although TLR2 gene was silenced by TLR2 siRNA, these results might be regulated by TLR4 promoter methylation and activated TLR4 mRNA level by 5-aza-dc in SNU-668 cells. TLR4 expression, like cell viability, is also upregulated in cells expressing TLR4 at a high level and in *TLR4* knockdown cells. These finding suggest that NF-κB signaling is blocked with BCL2 inhibition, triggering apoptosis, but the effect of combined treatment with 5-aza-dC and LPS also relatively repressed cell death with ABT-199. Our results suggest that epigenetic regulation of *TLR4* expression regulates NF-κB signaling *via* TLR4 (Figure 5C).

Next, to elucidate whether *TLR4* activation induced by 5-aza-dC and LPS affects NF-κB signaling, we investigated the effect of combined pretreatment with 5-aza-dC and LPS in gastric cancer cell lines expressing low and high levels of TLR4. We found significant time-dependent enhancement of p-IKKα/β level with LPS treatment of 5-aza-dC–pretreated SNU-638 and SNU-668 cells (Figure 5D). Then, to demonstrate the occurrence of epigenetic events at the *TLR4* promoter and NF-κB signaling through *TLR4* knockdown, we analyzed nuclear and cytosolic fractions of *TLR4* knockdown and control cells by western blotting (Figure 5D and 5E). First, to demonstrate NF-κB signaling induced by treatment with 5-aza-dC and LPS, proteins in the cytosolic fractions were quantified together with β-actin, We observed increased phosphorylation of IκBα in treated control siRNA-transfected cells In addition, ABT-199 treatment following LPS and 5-aza-dC pretreatment phosphorylated IκBα to a greater extent than treatment with ABT-199 alone, but to a lesser extent than combined 5-aza-dC and LPS treatment. Conversely, levels of phosphorylated IκBα were lower in *TLR4* knockdown cells than in control transfected cells. Moreover, Western analysis of nuclear fractions showed that H3, Sp1, and NF-κB were more inactivated in *TLR4* knockdown cells than in control transfected cells, but H3K4me3 and H3K9me3 levels were unchanged. On the basis of these results, we suggest that combined treatment with 5-aza-dC and LPS activates TLR4 signaling, which leads to phosphorylation of IKK α/β and IκBα and promotes nuclear translocation of NF-κB.

### *TLR4* expression induced by combined treatment with 5-aza-dC and LPS activates TLR4/NF-κB signaling

**Figure 6:**
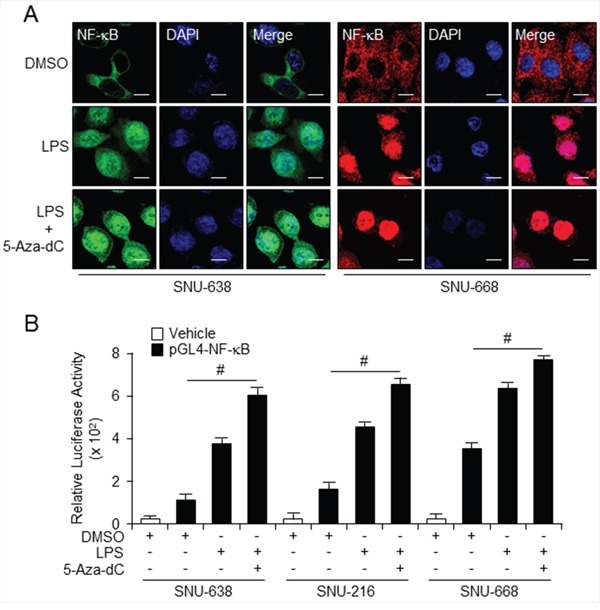
Combined treatment with LPS and 5-aza-dC activates TLR4/NF-κB signaling **A.** Immunofluorescence analysis using confocal microscopy of NF-κB in SNU638 cells stained with secondary antibody labeled with Alexa Fluor 488 (green) and SNU668 cells stained with secondary antibody labeled with Alexa Fluor 555 (red) after 3-d pretreatment with 5-aza-dC followed by 30-min incubation in LPS alone. Scale bar, 20 mm. **B.** Relative NF-κB expression in SNU638, SNU216, and SNU668 cells transfected with pGL4 (control) or pGL4 NF-κB-luc vector using Lipofectamine 2000, assessed by luciferase assay after 3-d incubation with LPS and 5-aza-dC alone and in the indicated combinations. Each samples were assayed in triplicate (# *p* < 0.01).

Previous studies suggest that co-expression of TLR4 with its ligand, LPS, induce activation of NF-κB in the tumor microenvironment, and also induce tumor growth and development [30,31]. Moreover, LPS-induced TLR4/NF-κB signaling allows nuclear translocation of NF-κB [32]. Our immunofluorescence data indicate that combined treatment with LPS and 5-aza-dC stimulates NF-κB in the cytosol and recruits it to the nucleus in SNU-638 and SNU-668 cells (Figure 6A). In addition, NF-κB translocates faster and to a greater extent in cells treated with LPS following pretreatment with 5-aza-dC than in cells treated with LPS alone. LPS and 5-aza-dC induction of NF-κB activity was investigated in gastric cell lines expressing TLR4 at low and high levels and transfected with the luciferase reporter construct pGL4-NFκB-luc (Figure 6B). NF-κB expression, demonstrated by luciferase activity, was increased in cells treated with LPS and 5-aza-dC compared with cells treated with DMSO alone. Although there was a strong change with LPS treatment, combination treatment with LPS and 5-aza-dC highly increased NF-κB activity and nuclear translocation in SNU-638 and SNU-216 which is low level of TLR4 expression rather than SNU-668 cells which is high TLR4 expression. These results are indicated that the low level of TLR4 expression which is hypermethylated condition may increase the demethylated effect of 5-aza-dC and NF-κB signal pathway than high level of TLR4 expression.

## DISCUSSION

In this study, our results suggested that TLR4 expression in gastric cancer correlated with tumor stages and difference of TLR4 expression related to epigenetic modification *via* DNA methylation. TLR4 is not only important in the regulation of immune responses to infection [33], but also is involved in noninfectious inflammatory diseases, such as tumor invasion and survival [34]. High level of TLR4 expression was found to be associated with increased incidence of metastasis and poor prognosis in breast carcinoma, colorectal cancer and hepatocellular carcinoma [35–37]. Although the expression of TLR4 in gastric cancer cells has been examined, the mechanisms and the molecular pathways mediated by TLR4 signaling in gastric tumorigenesis are still controversial. On the one hand, TLR4, as a negative regulator of cancer, inhibits tumor growth and progression. TLR4 expressed in cancer can prevent infection by microbial pathogens such as hepatitis B and C viruses [38,39], Helicobacter pylori [40,41], and human papilloma virus [42], which may cause cancer. High dose of LPS, TLR4 ligand, can directly kill tumor cells so that it has been used to treat colorectal and lung cancer in Phase II clinical trials [43]. TLR4 activation may also lead to tumor regression by increasing vascular permeability [44]. On the other hand, TLR4 stimulation promotes tumorigenesis. TLR4 upregulate the NF-κB signaling and produce anti-apoptotic proteins that promote carcinogenesis and cancer cell growth and proliferation [45,46]. When the function of TLR4 signaling pathways is gradually unveiled and confirmed in cancer cells, their regulation attracts much attention. In the past few decades, many regulators of TLR4 signaling pathways, including both positive and negative ones, have been found [47,48]. Understanding the precise mechanism of *TLR4* regulation is important to understanding the development and progression of gastric cancer. In these results, we found that *TLR4* expression was frequently upregulated in human gastric cancer tissues and cells such as AGS, SNU-216 and SNU-668, but was often downregulated in several gastric cancer cell lines such as NCI-N87, SNU-620, SNU-638, NUGC3 and MKN74. Different levels of *TLR4* expression commonly occurs by promoter methylation, and we also found that both MeCP2 and H3K9me3 contribute as repressors of *TLR4* expression, whereas transcription factor Sp1 and H3K4me3 participate as activators of *TLR4* transcription.

To further investigate the signal transduction and mechanism of TLR4 expression in various gastric cancer cells, the epigenetic modification of TLR4 has been characterized using 5-aza-dc in vitro. Epigenetic modifications, such as DNA methylation and histone modification, have been studied in various events in the cancer environment [49]. Recent reports demonstrate promoter methylation of tumor-related genes, including *MGMT*, *CDKN2B* and *RASSF1* [50]. DNA methyltransferase DNMT1 is important in regulation of gene expression, and DNMT1 inhibitor 5-aza-dC demethylates CpG sites via DNMT1 degradation [51]. In this study, we demonstrate that the methylation status of CpG sites on the *TLR4* promoter regulates transcription, and combination treatment of cells with 5-aza-dC and LPS induced TLR4/NF-κB signaling events related to critical regulators, including transcriptional repressor MeCP2 and transcriptional activator Sp1. MeCP2 expression was silenced through the interaction of transcriptional repression domain (TRD) with a corepressor, the Sin3A complex, which includes HDAC1 and HDAC2, and it was recently reported that MeCP2 is involved in histone modification *in vitro* and *in vivo* [52]. Moreover, Fuks et al. suggest that MeCP2-mediated methylation of the *H19* gene is specific for H3K9 [53]. TLR4-induced proinflammatory cytokine IL-6 is silenced by promoter methylation and binding of MeCP2 and H3K9me, and 5-aza-dC–induced reactivation of IL-6 induced NF-κB signaling [54].

Gastric cancer is a highly heterogeneous disease and necessary to figure out the alterations involved in individual gastric cancer so as to increase the chance to predict prognosis and establish effective treatment options. Gastric adenocarcinoma accounting for 90-95% of gastric cancers has two histological types- intestinal and diffuse types based on microscopic observation and growth patterns. They are widely different in their molecular pathogeneses [55]. Nonetheless, epigenetic modifications may play important role in the development of both types of gastric carcinomas. Activation of TLR4/NF-κB signaling in gastric cancer cell lines expressing TLR4 at low and high levels by the TLR4 ligand LPS and DNA methyltransferase inhibitor 5-aza-dC provides a new direction for research on TLR4 signaling in cells silenced by promoter methylation, and promote cell survival and proliferation [56]. Moreover, BCL2 inhibitor ABT-199 induced cell death in gastric cancer cell lines, but 5-aza-dC/LPS and ABT199 cotreatment relatively decreased cell death.

Recent studies have demonstrated that transcription factor Sp1 may bind the *TLR4* promoter [57]. Sp1 binding is commonly involved in the demethylation of CpG sites on various gene promoters [58]. When we carried out ChIP with a Sp1 antibody, Sp1 binding increased to a greater extent in SNU-668 cells, which express TLR4 at a high level, compared to SNU-638 cells which express TLR4 at a low level, and also decreased Sp1 levels of nuclear fraction in *TLR4* knockdown cells. Here, Sp1 binding suppressed CpG methylation, and Sp1-binding–induced demethylation plays a role as an activator of *TLR4* transcription. MeCP2 was first shown to bind methylated DNA and is an epigenetic transcriptional repressor that inhibits gene expression through the interpretation of DNA methylation and histone modification [59]. DNA methylation is frequently associated with gene silencing in various tumors, and MeCP2 may also be a potential regulator of tumorigenesis [60]. MBD proteins bind to methylated cytosines and interact with corepressors, including HDACs [61]. This complex of MeCP2, HDACs, and methylated DNA represents an important mechanism of gene silencing and chromatin remodeling [62]. Moreover, methylation at lysine 9 of histone H3 (H3K9) and DNA methylation are associated with MeCP2, and highlight MeCP2-associated histone modification for transcriptional repression [63].

In summary, by promoting rounds of histone and DNA methylation, MeCP2 binding of the *TLR4* promoter maintains the hypermethylation status of CpG sites and repressor-mediated silencing of *TLR4* expression, but Sp1 binding induced *TLR4* activation from a hypomethylated promoter in cells expressing *TLR4* at a high level, and LPS and 5-aza-dC also induced TLR4/NF-κB signaling. Our findings provide basic information for the different expression of TLR4 *via* epigenetic modification of TLR4 promoter in various gastric cancer cells and the relationship between epigenetic modification and TLR4 expression may contribute to this regulation of these alterations results in enhanced levels of TLR4 that can confer responsiveness to anti-tumor drug.

## MATERIALS AND METHODS

### Genomic DNA extraction from gastric tissues

Post-surgical samples of gastric carcinoma and nearby normal tissues were obtained from Samsung medical hospital in Seoul, Korea. A few milligrams of tumor and adjacent normal tissue were taken and genomic DNA and RNA was isolated using a genomic DNA purification kit (Promega, Madison, WI, USA) and Trizol (Invitrogen, Carlsbad, CA, USA) according to the manufacturer's protocols.

### Cell culture

Human gastric cancer cell lines, AGS, SNU-216, NCI-N87, SNU-620, SNU-638, MKN74 and SNU-668, were obtained from the Korean Cell Line Bank (Seoul, Korea) and grown in Dulbecco modified Eagle medium (Gibco-BRL, Grand Island, NY) supplemented with heat-inactivated 10% fetal bovine serum (Gibco-BRL) and antibiotics (100 U/mL Penicillin and 100 mg/mL streptomycin, Gibco-BRL). Cells were maintained at 37°C in a humidified, 5% CO_2_/air atmosphere. To identify regulation by methyltransferase, 5′-Aza-2′-deoxycytidine (Sigma, St. Louis MO, USA), a methyltransferase inhibitor, was added to the culture medium 1 μM for 72 hrs, respectively, to induce demethylation of the cytosine residues.

### Sodium bisulfite modification and methylation-specific PCR

Chromosomal DNA was isolated from the cell cultures in a 75 cm^2^ culture flask using a genomic DNA purification kit (Promega, Madison, WI) according to the manufacturer's protocol. The extracted DNA was eluted with 250 μl of distilled water. Sodium bisulfite modification of genomic DNA was carried out using an EZ DNA Methylation Kit (Zymo Research, USA) according to the manufacturer's protocol using 0.1 mg of purified DNA. Primers used in this study were as follows: methylated *TLR4* (sense, 5′-GTT TAG CGG TTT ACA TGA TTT GAT-3′, antisense, 5′-CCC GCC CCT TCT TAT AAA AAA ACT-3′); unmethylated *TLR4* (sense, 5′-GTT TAG TGG TTT ATA TGA TTT GAT-3′, antisense, 5′-CCC ACC CCT TCT TAT AAA AAA ACT-3′). Real-time Methylation-Specific PCR (MSP) was performed using a Power SYBR Green Kit (Applied Biosystems, Foster City, CA, USA) according to the manufacturer's protocol. A methylation index was calculated for each sample using the following formula: methylation index = 1 / [1 + 2^−(CTu − CTme)^] × 100%, where CTu is the average cycle threshold (CT) obtained from duplicate quantitative PCR analyses using the unmethylated primer pair, and CTme is the average CT obtained using the methylated primer pair.

### Quantitative and reverse transcription PCR

Total RNA from cell culture was prepared using Trizol reagent according to the manufacturer's protocols (Invitrogen, Carlsbad, CA, USA). Reverse transcription was conducted using 10 μg of total RNA with a reverse transcription kit (Promega). Expression levels of selected genes were measured by quantitative PCR (qPCR) analysis in order to confirm consistency with those from the microarray data. Primers used in this study were as follows: *TLR4* for qPCR (sense, 5′-GCT TAT CTG AAG GTG TTG CA-3′, antisense, 5′-CAG AGT TTC CTG CAA TGG AT-3′); *TLR2* for qPCR (sense, 5′-GGC CAG CAA ATT ACC TGT GT-3′, antisense, 5′-AGG CGG ACA TCC TGA ACC T-3′); TLR4 for reverse transcription PCR (sense, 5′-TTG GGA CAA CCA GCC TAA AG-3′, antisense, 5′-TGC CAT TGA AAG CAA CTC TG-3′); TLR2 for reverse transcription PCR (sense, 5′-GGC CAG CAA ATT ACC TGT GT-3′, antisense, 5′-ATA CCA CAG GCC ATG GAA AC-3′); *ACTB* (sense, 5′-GAG ACC TTC AAC ACC CCA GCC-3′, antisense, 5′-GGA TCT TCA TGA GGT AGT CAG-3′); *GAPDH* (sense, 5′-CAT GAC CAC AGT CCA TGC CAT-3′, antisense, 5′-AAG GCC ATG CCA CTG AGC TTC-3′). *ACTB and GAPDH* were used as reaction controls. 1 ml of cDNA was used for the PCR, and triplicate reactions were performed for each sample using a Power SYBR Green Kit (Applied Biosystems, Foster City, CA) with gene-specific primers on an ABI StepOnePlus instrument (Applied Biosystems). RNA quantity was normalized to β-actin and GAPDH content, and gene expression was quantified according to the 2^−ΔCt^ method.

### Transfection

SNU-638 and SNU-668 cells (2 × 10^5^ or 1 × 10^6^ cell/well) in 24- or 6-well plate were transfected with these double-stranded siRNAs (30 nmol/ml) of MeCP2, Sp1, TLR2 and TLR4 (Bioneer) for 24 hr by the Lipofectamine 2000 (Invitrogen) method and recovered in RPMI1640 medium (Welgene) containing 10% fetal bovine serum for 24 hrs.

### Western blot analysis

Cells resuspended RIPA lysis buffer (50 mmol/L Tris-HCl, pH7.4, 150 mmol/L NaCl, 1% NP40, 0.25% Na-deoxycholate,1 mmol/L phenylmethylsulfonylfluoride (PMSF), 1 mmol/L sodium orthovanadate, 1X sigma protease inhibitor cocktail). Protein was measured using a BCA Protein Assay Kit (Pierce, Rockford, USA). Proteins were size-fractionated by PAGE and transferred to PVDF membrane. Nonspecific binding was blocked by incubation with PBS-T including 5% powdered milk and 1% Triton X-100. Membranes were incubated overnight at 4°C with primary antibodies (1:1,000) including Sp-1, MeCP2, IκBa, p-IκBa (Santa cruz) and H3K4me3, H3K9me3, H3 (Millipore) and p-IKKα/β (Cellsignaling) followed by incubation with polyclonal HRP-conjugated secondary antibodies (Sigma, 1:6,000) for 1 hour at room temperature. Membranes with any HRP-conjugate secondary antibody provided by Clarity Western ECL substrate (Bio-rad, USA) and the protein bands were visualized by exposure to X-ray film.

### Chromatin immunoprecipitation(ChIP) assay

ChIP assays were performed using an EZ ChIP Chromatin Immunoprecipitation kit (Millipore, Billerica, MA, USA) as described in the supplier's protocol. Briefly, the cross-linked chromatin was sonicated after cell lysis and then incubated with antibodies against HDAC1, HDAC2, HDAC6, SIRT1, SIRT2, SIRT3, MBD1, MeCP2, Sp-1 (Santa Cruz Biotechnology) and H3K4monomethylation (H3K4me1), H3K4dimethylation (H3K4me2), H3K4trimethylation (H3K4me3), H3K9trimethylation (H3K9me3), H3K27trimethylation (H3K27me3) (Millipore) at 4°C overnight. The immunocomplex was precipitated with Protein A-agarose (Millipore), and the beads were washed, sequentially treated with 10 μl of RNase A (37°C for 30 min) and 75 μl of Proteinase K (45°C for 4 h), and incubated at 65°C overnight to reverse cross-link the chromatin. The DNA was recovered by phenol-chloroform extraction and coprecipitation with glycogen, and dissolved in 50 μl of Tris-EDTA (TE) buffer. DNA was amplified by PCR using 1 μl of the precipitated DNA. PCR primers as follows: *TLR4* (sense, 5′-GTG AGT TTC TTC ACA AGA AGG G-3′, antisense, 5′-GGA GAG AGA GCC TTG AAA GAG G-3′) were designed to amplify the putative Sp-1 binding sites at the TLR4 gene promoter. Quantitative PCR conditions were 40 cycles at 94°C for 40 s, 60°C for 1 min, and 72°C for 40s.

### Nuclear and cytoplasmic fractionation

Fractionation of cytoplasmic and nuclear extracts was carried out using Nuclear Extract kit (Active motif) according to manufacturer's instructions. Supernatants used as cytoplasmic fraction, whereas pellets were resuspended in 50 ml of complete lysis buffer and supernatants were used as nuclear fractions by centrifuging at 14,000 × *g* for 10 minutes at 4°C.

### Tissue microarray

Tissue microarray slides for human gastric normal and tumor were purchased from SuperBioChips (SuperBioChips Laboratories, Seoul, Korea). Tissue sections were deparaffinized by xylene and blocked by normal serum. Primary antibody was diluted with anti-TLR4 (Santa Cruz Biotechnology, Inc.) antibody with CAS blocking solution (invitrogen) for overnight. And slides incubated with biotinylated secondary antibody for overnight. Using DAB substrate kit (DAB substrate kit, vector laboratories), tissue slides stained with substrate at room temperature until suitable staining develops immunohisto- chemistry staining was performed as the manufacturer recommended (VECTASTAIN ABC KIT, Vector laboratories).

### Immunofluorescence

After 5′-Aza-dC and LPS combination treatment, SNU-638 and SNU-668 were fixed for 15 min with 4% paraformaldehyde and stained by cell staining for immunofluorescence microscopy method according to the manufacturer's protocol (Life technology). The slides were incubated with blocking buffer (1% BSA in PBS) for 30 min at 37°C and exposed overnight to anti-NF-κB (Santa Cruz) antibody (1:200 dilution in blocking buffer) at 4°C. Secondary antibody was stained using red-fluorescent Alexa Fluor 555 goat anti-rabbit and -mouse, and green-fluorescent Alexa Fluor 488 goat anti-rabbit and -mouse. Nuclear DNA was stained with DAPI (Sigma). Samples were analyzed with a ZEISS LSM 510 META confocal microscope.

### Cell viability assay

WST-1 assay was performed according to the manufacturer's instructions (Roche, Mannheim, Germany) with 30 μl of WST-1 reagent was added to each well of a 24-well plate (1 × 10^4^ cell/well). After 1h of incubation using CO_2_ incubator, the conversion of WST-1 reagent into chromogenic formazan was monitored with a spectrophotometer. On day 1 after plating, cells were treated with methyltransferase inhibitor, 5′-Aza-2′-deoxycytidine (Sigma, St. Louis MO, USA), 1 μM for 72 hr to induce demethylation of the cytosine residues, respectively and also added Bcl2 inhibitor, ABT-199 (Abmole BioScience), 1 μM for 24 hr. LPS (Sigma, St. Louis MO, USA) was applied to the gastric cancer cells at concentrations of 10 mg/ml.

### Luciferase assay

pGL4.32 (luc2P/NF-κB-RE/Hygro) vector (pGL4NFkB-luc) and pGL4.74 (hRluc/TK) vector (pGL4-Renilla) were purchased from Promega (Madison, WI, USA). SNI-638, SNI-216 and SNU-668 cells at 70% confluence in 6-well plates were transfected with reporter luciferase plasmid, pGL4 basic, pGL4-Renilla and pGL4NFkB-luc, using Lipofectamine 2000 (Invitrogen) according to the manufacturer's instructions, respectively. Luciferase activity was measured 36 hours after transfection in three independent cultures using a dual-luciferase reporter assay kit (Promega) on Molecular Devices Filter Max F3 (Sunnyvale, CA, USA).

### Statistical analysis

Student's *t*-test was used to detect differences in the methylation and expression level between normal and cancerous tissues. *P*-values < 0.05 were considered significant.

## SUPPLEMENTARY FIGURES


