# Supplementary material for: Epigenetic modification of TLR4 promotes activation of NF-κB by regulating methyl-CpG-binding domain protein 2 and Sp1 in gastric cancer
Source: Oncotarget. 2015 Dec 10;7(4):4195–209. doi: 10.18632/oncotarget.6549 (PMC4826199; doi:10.18632/oncotarget.6549)
Supplement: Supplementary file 1 [file oncotarget-07-4195-s001.pdf]

SUPPLEMENTARY FIGURES

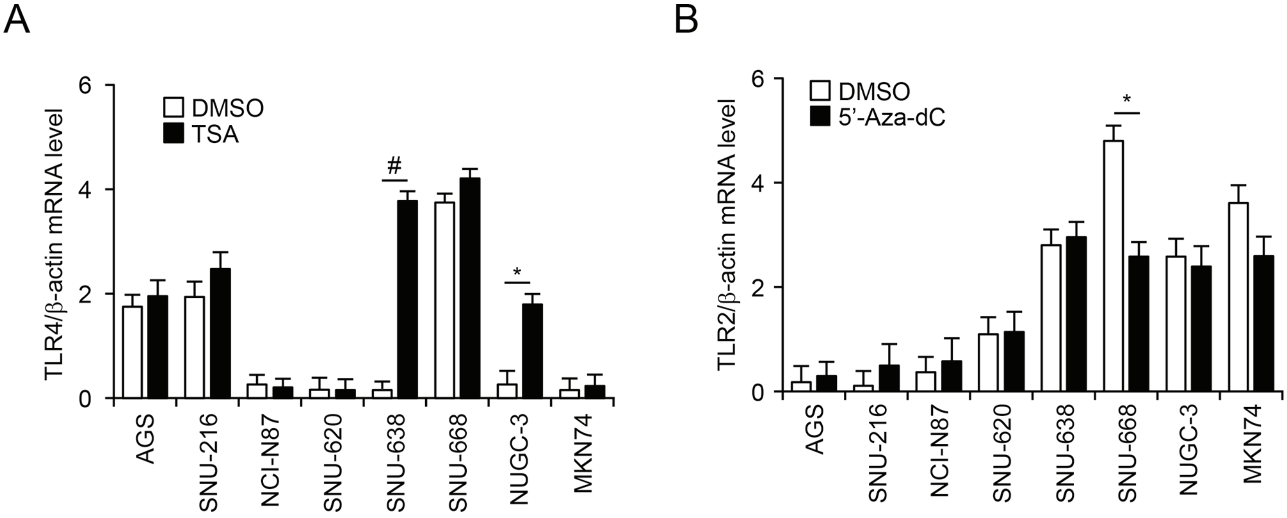

Supplementary Figure S1: Expression levels of *TLR2* and *TLR4* mRNA in gastric cell lines treated with 5-aza-dC and trichostatin A. A. qPCR analysis of relative TLR4 mRNA level in the indicated control (DMSO)- and trichostatin A (TSA)-treated gastric cell lines. **B.** qPCR analysis of relative *TLR2* mRNA level in the indicated control (DMSO)- and 5-aza-dC–treated gastric cell lines. *$p<0.05$, #$p<0.01$.

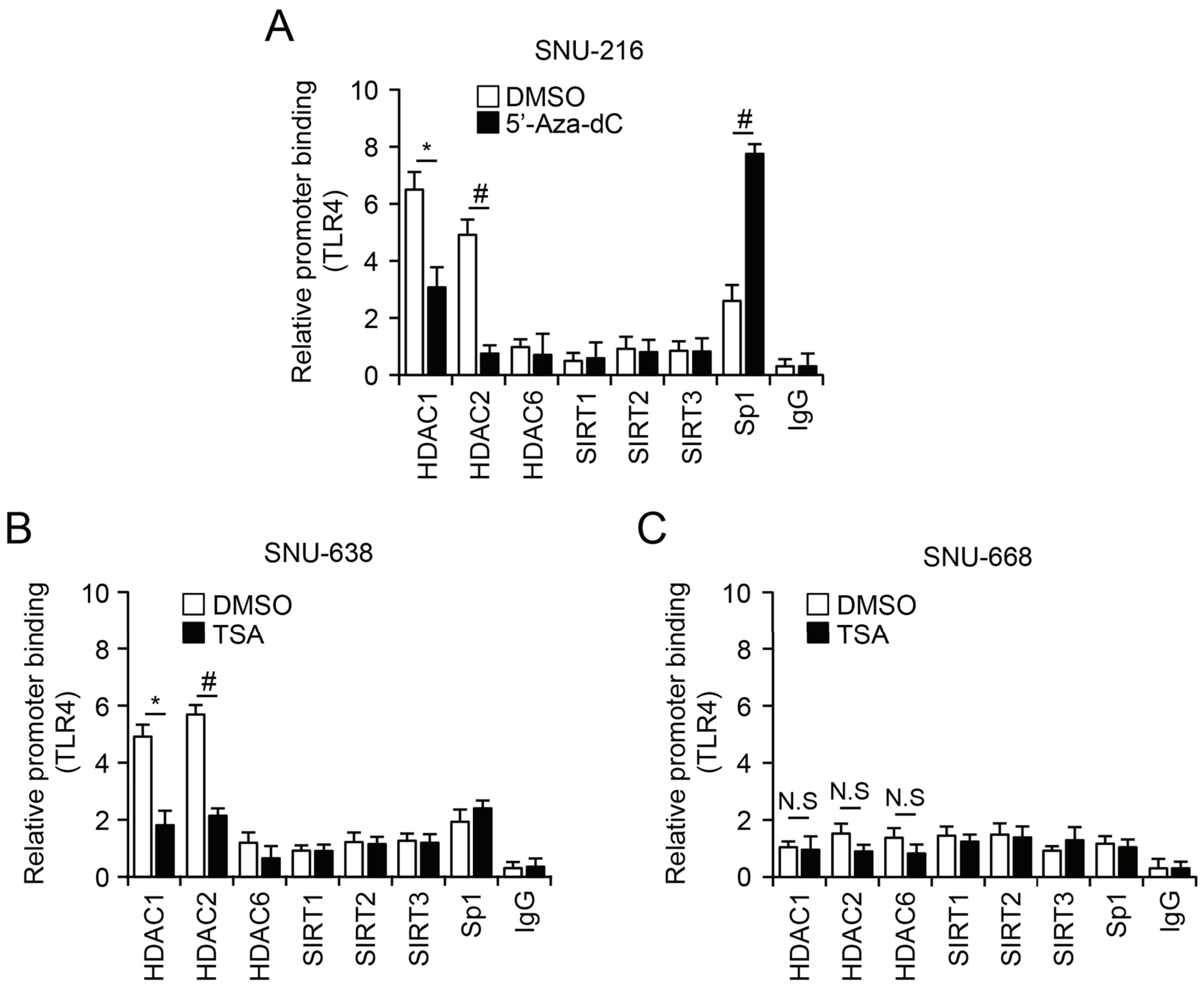

Supplementary Figure S2: Sp1 and HDACs bind to *TLR4* promoter in TSA-treated gastric cell lines. A–C. Results of chromatin immunoprecipitation (ChIP) of lysates of the indicated gastric cell lines treated with 5-aza-dC (A) and TSA (B and C) using IgG (control) and antibodies against Sp1, HDAC1, HDAC2, HDAC6, SIRT1, SIRT 2, and SIRT3. *p<0.05, #p < 0.01.

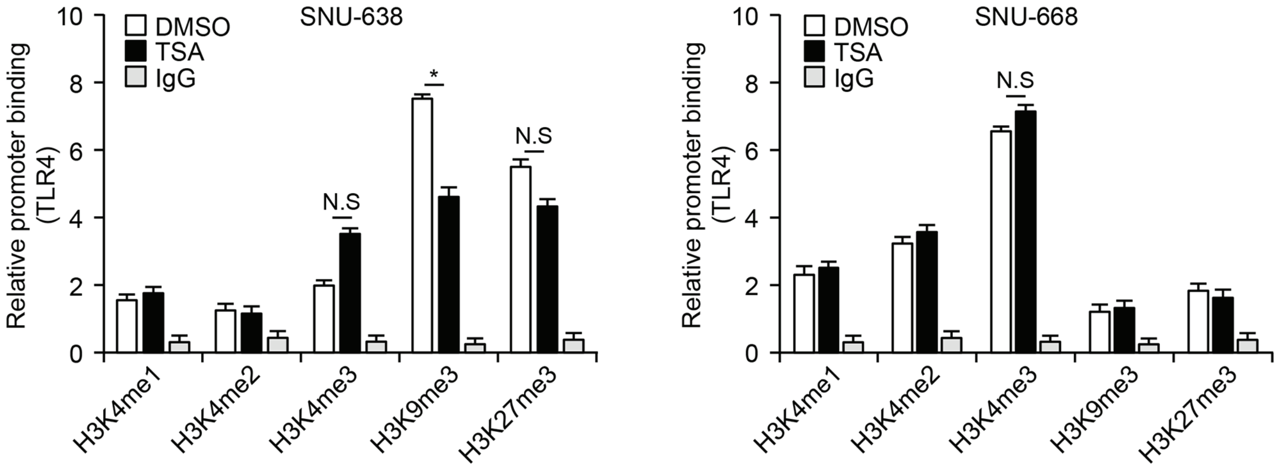

Supplementary Figure S3: Trimethylated histones bind to *TLR4* promoter in TSA-treated gastric cell lines. Results of ChIP of lysates of SNU-638 (left) and SNU-668 (right) cells treated with DMSO (control) or TSA by IgG (control) and antibodies against the indicated methylated histones. *$p<0.05$

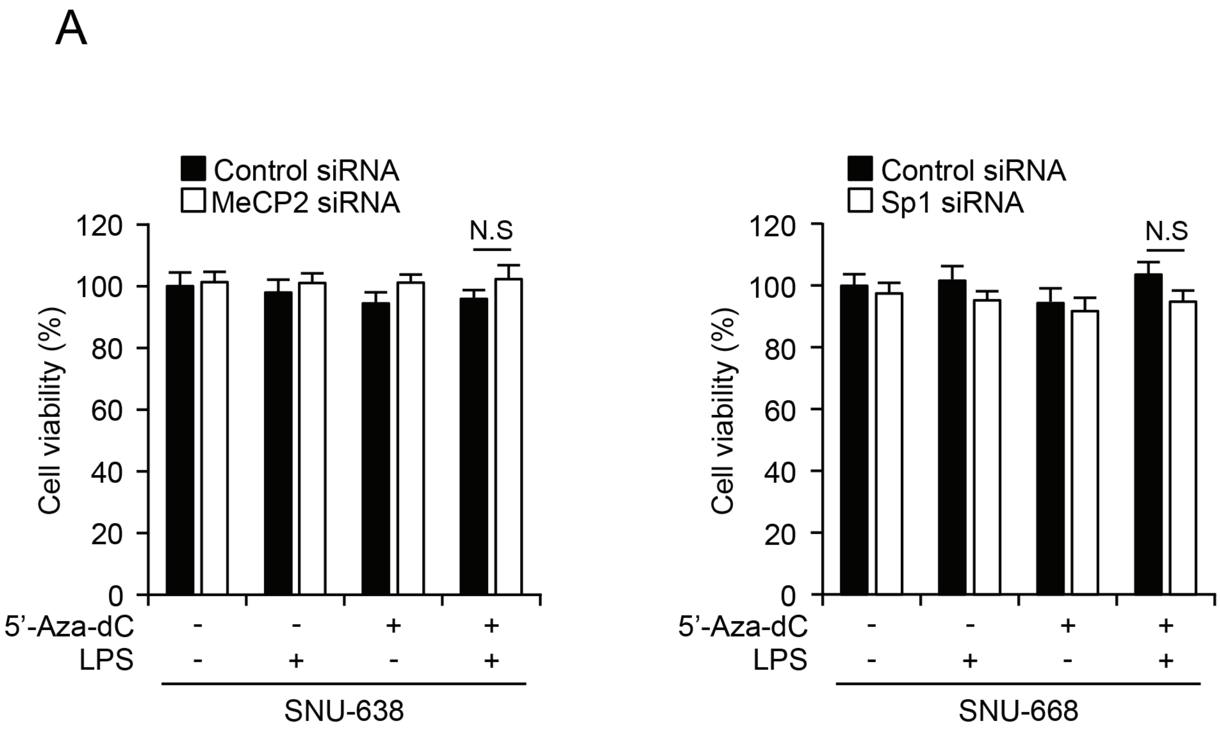

Supplementary Figure S4: Knockdown of Sp1 and MeCP2 did not affect viability of gastric cells lines treated with LPS and/or 5-aza-dC. Viability of control and MeCP2 knockdown SNU-638 (left) and control and Sp1 knockdown SNU-668 (right) cells treated with 5-aza-dC and LPS alone or together.

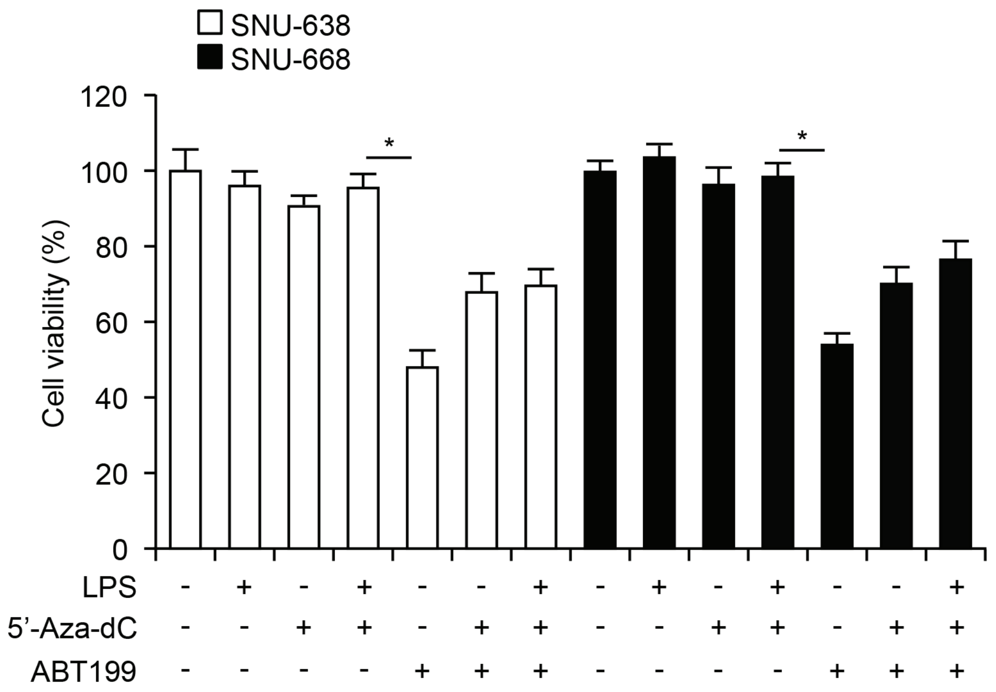

Supplementary Figure S5: Viability of SNU638 and SNU668 cells treated with LPS, 5-aza-dC, and ABT-199 alone and together.
